# The PedsQL™ as a patient-reported outcome in children and adolescents with fibromyalgia: an analysis of OMERACT domains

**DOI:** 10.1186/1477-7525-5-9

**Published:** 2007-02-12

**Authors:** James W Varni, Tasha M Burwinkle, Christine A Limbers, Ilona S Szer

**Affiliations:** 1 Department of Pediatrics, College of Medicine, Department of Landscape Architecture and Urban Planning, College of Architecture, Texas A&M University, 3137 TAMU, College Station, TX 77843-3137, USA; 2 The Children's Hospital at Scott & White, Department of Pediatrics, College of Medicine, Texas A&M University Health Science Center, 2401 South 31^st ^Street, Temple, TX 76508, USA; 3 Department of Psychology, College of Liberal Arts, Texas A&M University, College Station, TX 77843-3137, USA; 4 Division of Rheumatology, Rady Children's Hospital and Health Center, Department of Pediatrics, University of California, School of Medicine, San Diego, CA 92123, USA

## Abstract

**Background:**

Fibromyalgia is a chronic health condition characterized by widespread musculoskeletal pain, multiple tender points on physical examination, generalized muscular aching, stiffness, fatigue, nonrestorative sleep pattern, cognitive dysfunction, and mood disturbance. Recently, the Outcome Measures in Rheumatoid Arthritis Clinical Trials (OMERACT) Fibromyalgia Syndrome Workshop ranked and prioritized the domains that should be consistently measured in fibromyalgia clinical trials, specifically, pain, generic health-related quality of life, fatigue, sleep quality, and physical function. The focus of these deliberations was exclusively on adult patients, and to our knowledge, these domains have not been previously tested within a multidimensional framework in children and adolescents with fibromyalgia.

**Methods:**

An analysis to determine the feasibility, reliability, and validity of the PedsQL™ 4.0 (Pediatric Quality of Life Inventory™) Generic Core Scales, PedsQL™ Multidimensional Fatigue Scale, and PedsQL™ Rheumatology Module Pain and Hurt Scale as patient-reported outcome (PRO) measures for pediatric patients with fibromyalgia. The PedsQL™ Scales were completed by 59 families in a pediatric rheumatology clinic in a large children's hospital.

**Results:**

The PedsQL™ evidenced minimal missing responses (0.53% patient self-report, 0.70% parent proxy-report), achieved excellent reliability for the Generic Core Scales Total Scale Score (α = 0.88 patient self-report, 0.87 parent proxy-report), the Multidimensional Fatigue Scale Total Scale Score (α = 0.94 patient self-report, 0.94 parent proxy-report), and acceptable reliability for the 4-item Rheumatology Module Pain and Hurt Scale (α = 0.68 patient self-report, 0.75 parent proxy-report). The PedsQL™ Generic Core Scales and Multidimensional Fatigue Scale significantly distinguished between pediatric patients with fibromyalgia and healthy children. Pediatric patients with fibromyalgia self-reported severely impaired physical and psychosocial functioning, significantly lower on most dimensions when compared to pediatric cancer patients receiving cancer treatment, and significantly lower on all dimensions than pediatric patients with other rheumatologic diseases. Patients with fibromyalgia self-reported significantly greater pain and fatigue than pediatric patients with other rheumatologic conditions, and generally more fatigue than pediatric patients receiving treatment for cancer.

**Conclusion:**

The results demonstrate the excellent measurement properties of the PedsQL™ Scales in fibromyalgia. These PedsQL™ Scales measure constructs consistent with the recommended OMERACT Fibromyalgia Syndrome Workshop domains. The findings highlight the severely impaired HRQOL of pediatric patients with fibromyalgia. Regular monitoring of pediatric patients with fibromyalgia will help identify children and adolescents at risk for severely impaired HRQOL. These PedsQL™ Scales are appropriate outcome measures for clinical trials and health services research for pediatric patients with fibromyalgia.

## Background

Fibromyalgia (FM) is a chronic health condition characterized by widespread musculoskeletal pain, multiple tender points on physical examination, generalized muscular aching, stiffness, fatigue, nonrestorative sleep pattern, cognitive dysfunction, and mood disturbance [1-3]. FM is considered a clinical syndrome presumably related to central neuromodulatory dysregulation [4]. The treatment of FM is complicated by the fact that there are no objective findings on the physical examination or laboratory tests that, in other rheumatologic conditions, confirm the extent of disease severity and aid in the establishment of a diagnosis. Consequently, the diagnosis of FM is based on illness history, exclusion of other causes of symptoms, verbal self-report, and physical examination [1].

Yunus and Masi were the first to describe the juvenile primary fibromyalgia syndrome (JPFS) in pediatric patients [5]. Consistent with the literature regarding adult patients [6], FM in pediatric patients is more common in girls than boys [5]. Although there are limited epidemiological data about the prevalence of FM in children and adolescents, it accounts for approximately 7–8% of new patient diagnoses in the pediatric rheumatology clinical practice, with estimates of population-based schoolchildren prevalence studies ranging from 1.2% to 7.5% [7].

The lack of physiological markers of disease activity for FM complicates the clinical decision-making process, since the treating physician cannot monitor the course of the illness with objective disease indicators that are available for other rheumatologic diseases such as juvenile idiopathic arthritis. Given the lack of objective outcomes measures, and the emerging therapies currently being tested for FM, the need for reliable and valid patient-reported outcome instruments for FM, including health-related quality of life instruments, has become urgent [4].

### Health-related quality of life assessment in fibromyalgia

Health-related quality of life (HRQOL) has been progressively acknowledged as an essential health outcome measure in clinical trials and health services research and evaluation [8-10]. A HRQOL instrument must be multidimensional, consisting at the minimum of the physical, psychological (including emotional and cognitive), and social health dimensions delineated by the World Health Organization [11,12]. Studies with adult patients with FM have demonstrated that in comparison to healthy controls, patients with FM report substantially lower HRQOL across multiple domains [13-15].

### Health-related quality of life assessment in pediatric patients

Although the measurement of HRQOL in pediatric clinical trials has been advocated for a number of years [16], the emerging paradigm shift toward patient-reported outcomes (PROs) in clinical trials [12] has provided the opportunity to further emphasize the value and essential need for pediatric patient self-report measurement as efficacy outcomes in clinical trials for pediatric chronic health conditions [17-20]. By definition, patient-reported outcomes (PROs) are self-report instruments that directly measure the patient's perceptions of the impact of disease and treatment as clinical trial endpoints [12]. PROs include multi-item HRQOL instruments, as well as single-item symptom measures (e.g., pain intensity visual analogue scale [VAS]) [21-23].

It is well documented in both the adult and pediatric literature that information provided by proxy-respondents is not equivalent to that reported by the patient [24,25]. Imperfect agreement between self-report and proxy-report, termed cross-informant variance [26], has been consistently documented in the HRQOL measurement of children with chronic health conditions and healthy children [27-34].

While pediatric patient self-report should be considered the standard for measuring perceived HRQOL [35], there may be circumstances when the child is too young, too cognitively impaired, too ill or fatigued to complete a HRQOL instrument, and parent proxy-report may be needed in such cases [36]. Further, it is typically parents' perceptions of their children's HRQOL that influences healthcare utilization [37-39]. Thus, HRQOL instruments should be selected that measure the perspectives of *both *the child and parent since these perspectives may be independently related to healthcare utilization, risk factors, and quality of care [40].

Ideally, parent and child HRQOL instruments should measure the same constructs with parallel items in order to make comparisons between self and proxy report more meaningful [41,42]. Even when children are able to self-report, parent proxy-report should be considered as a secondary outcome measure given parents' expanding role in clinical decision-making and home treatment regimens for pediatric chronic health conditions [40]. Thus, there is a clear and defined role for parent proxy-report instruments in the assessment of pediatric HRQOL outcomes.

### OMERACT Fibromyalgia Syndrome Workshop domains

Recently, the Outcome Measures in Rheumatoid Arthritis Clinical Trials (OMERACT) Fibromyalgia Syndrome Workshop ranked and prioritized the domains that should be consistently measured in FM clinical trials in adults, specifically, pain, generic HRQOL, fatigue, sleep quality, and physical function [4]. To our knowledge, these domains have not been previously tested within a multidimensional framework in children and adolescents with FM.

Consequently, the objectives of the current analyses are to determine the feasibility, reliability and validity of pediatric patient self-report and parent proxy-report for children and adolescents with FM utilizing the PedsQL™ 4.0 Generic Core Scales, PedsQL™ Multidimensional Fatigue Scale, and PedsQL™ Rheumatology Module Pain and Hurt Scale. These scales were selected for this investigation since they measure the primary symptom domains identified by the OMERACT Fibromyalgia Syndrome Workshop [4].

Based on the extant literature on HRQOL in pediatric chronic health conditions in general and FM in particular, we hypothesized that pediatric patients with FM would self-report significantly lower physical and psychosocial functioning and greater fatigue than healthy children. We further examined the agreement between pediatric patient self-report and parent proxy-report, expecting moderate agreement based on the extant literature [43] and previous research with the PedsQL™ in pediatric chronic health conditions [44-46]. In order to further determine the clinical magnitude of the hypothesized negative impact of FM on pediatric patient self-reported HRQOL, pain, and fatigue, we conducted comparative analyses between pediatric patients with FM and pediatric cancer patients receiving cancer treatment (chemotherapy and radiation therapy) and pediatric patients with other rheumatologic conditions, both groups who have previously demonstrated impaired self-reported HRQOL, pain, and fatigue using the PedsQL™ [44-46].

## Method

### Participants and settings

#### Pediatric fibromyalgia sample

Participants were 59 children and adolescents ages 8 to 18 years, and their parents (n = 57), diagnosed with FM by their pediatric rheumatologist using the Yunus & Masi criteria [5]. Pediatric patients with FM were identified through the appointment schedule at the Pediatric Rheumatology Clinic at Rady Children's Hospital and Health Center, San Diego. Parents of children identified as possible study participants were informed of the study after checking in for their appointment, but before being seen by their healthcare provider. Written parental informed consent and child assent were obtained.

Patients and parents completed the PedsQL™ 4.0 Generic Core Scales and PedsQL™ 3.0 Rheumatology Module Pain and Hurt Scale during a routine rheumatology clinic visit. The PedsQL™ Multidimensional Fatigue Scale was administered to patients (n = 29) and parents (n = 26) beginning approximately midway through participant recruitment since it was still under development at the initiation of this field test in FM. The PedsQL™ was self-administered for both children and their parents. Parents and children completed the PedsQL™ separately. One parent (79.7% mothers; 8.5% fathers; 5.1% other, 6.8% missing) completed the proxy-report version. Trained clinic personnel were available to answer questions regarding the self-administered instruments after the instructions had been given and clarified. This research protocol was approved by the Institutional Review Board at Children's Hospital and Health Center, San Diego.

Although the PedsQL™ can be administered for children ages 2–18, children in this sample were all between the ages of 8 years and 18 years given that it is relatively uncommon for young children to have fibromyalgia. The average age of the 53 girls (89.8%) and 6 boys (10.2%) was 13.73 years (SD= 2.92). With respect to race/ethnicity, the sample contained 35 (59.3%) White non-Hispanic, 8 (13.6%) Hispanic, 2 (3.4%) Black non-Hispanic, 2 (3.4%) American Indian or Alaskan Native, 1 (1.7%) other, and 11 (18.6%) missing. Mean socioeconomic status (SES) was 46.59 (SD = 12.20), based on the Hollingshead index, indicating on average a middle-class family SES [47].

#### Pediatric cancer sample

The pediatric cancer sample consisted of children and adolescents ages 8 to 18 years receiving cancer treatment (chemotherapy and radiation therapy) and their parents who were randomly matched by age group to the FM sample utilizing the SPSS Version 14.0 statistical software random sample case selection command [48]. These participants, derived from the PedsQL™ 3.0 Cancer Module field test [44], were recruited from the Hematology/Oncology Centers at Rady Children's Hospital and Health Center, San Diego and Childrens Hospital Los Angeles. Written parental informed consent and child assent were obtained. The PedsQL™ was self-administered for both children and their parents. The sample included children with acute lymphocytic leukemia (n = 38, 46.9%), brain tumor (n = 8, 9.9%), Hodgkin's lymphoma (n = 6, 7.4%), non-Hodgkin's lymphoma (n = 5, 6.2%), Wilm's tumor (n = 1, 1.2%), and other cancers (n = 23, 28.4%). For all forms combined, the average age of the 39 boys (48.1%) and 41 girls (50.6%; Missing = 1, 1.2%) was 12.88 years (SD = 3.28). With respect to race/ethnicity, the sample contained 39 (48.1%) Hispanic, 30 (37.0%) White non-Hispanic, 3 (3.7%) Black non-Hispanic, 1 (1.2%) Asian/Pacific Islander, 1 (1.2%) American Indian or Alaskan Native, 6 (7.4%) other, and 1 (1.2%) missing. Mean socioeconomic status (SES) was 34.74 (SD = 15.43), based on the Hollingshead index, indicating on average a lower middle-class family SES [47].

#### Pediatric rheumatology sample

The rheumatology sample consisted of children ages 8 to 18 years and their parents who were randomly matched by age group to the FM sample utilizing the SPSS Version 14.0 statistical software random sample case selection command [48]. These participants, derived from the PedsQL™ 3.0 Rheumatology Module field test [45] and the PedsQL™ Multidimensional Fatigue Scale field test in pediatric rheumatology [46], were recruited from the Pediatric Rheumatology Clinic at Rady Children's Hospital and Health Center, San Diego. Written parental informed consent and child assent were obtained. The PedsQL™ was self-administered for both children and their parents. For all forms combined, the average age of the 42 boys (26.8%) and 114 girls (72.6%; Missing = 1, 0.6%) was 13.43 years (SD = 3.32). With respect to race/ethnicity, the sample contained 64 (40.8%) White non-Hispanic, 30 (19.1%) Hispanic, 8 (5.1%) Asian/Pacific Islander, 6 (3.8%) Black non-Hispanic, 2 (1.3%) American Indian or Alaskan Native, 3 (1.9%) other, and 44 (28.0%) missing. Mean socioeconomic status (SES) was 46.79 (SD = 14.63), based on the Hollingshead index, indicating on average a middle-class family SES [47].

#### Healthy children sample: Generic Core Scales

Participants included children and adolescents ages 8 to 18 years and their parents who were randomly matched by age group and gender to the FM sample utilizing the SPSS Version 14.0 statistical software random sample case selection command [48]. "Healthy" children and adolescents, defined as children and adolescents without parent-identified chronic illness or disability, were derived from the PedsQL™ 4.0 Generic Core Scales Database^SM ^and were recruited from either a California State Children's Health Insurance Program (SCHIP) (n= 1758; 51.4%) [49] or from a school-based study within the San Diego Unified School District (n = 1662; 48.6%) [50]. Children and parents completed the PedsQL™ 4.0 Generic Core Scales, which was self-administered for both children and their parents. For all forms combined, the average age of the 342 boys (10.0%) and 3078 girls (90.0%) was 11.54 years (SD = 2.44). The sample was heterogeneous with respect to race/ethnicity with 1521 (44.5%) Hispanic, 790 (23.1%) Asian/Pacific Islander, 553 (16.2%) White non-Hispanic, 288 (8.4%) Black non-Hispanic, 11 (0.3%) American Indian or Alaskan Native, 54 (1.6%) other, and 203 (5.9%) missing. The statewide SCHIP sample and large metropolitan school district sample were representative of low to low-middle income families.

#### Healthy children sample: Multidimensional Fatigue Scale

Participants were healthy children ages 5 to 18 years and parents of healthy children ages 2 to 18 years derived from the PedsQL™ Multidimensional Fatigue Scale field test in pediatric rheumatology [46]. Participants were not randomly matched by age and gender to the FM sample given the small healthy children sample available. This healthy sample was recruited at the Orthopedic Clinic in Rady Children's Hospital and Health Center, San Diego, and included patients who presented with broken bones or fractures 6 months prior to assessment with the PedsQL™. All patients were identified by the clinic nurse as being healthy at the time of assessment (e.g., no current problems due to their orthopedic injury). Participants were administered the PedsQL™ Multidimensional Fatigue Scale Acute Version (7 day recall period) via telephone, after obtaining informed consent and assent. For all forms combined, the average age of the 69 boys (67.6%) and 30 girls (29.4%; Missing = 3, 2.9%) was 9.89 years (SD = 5.02). The sample was heterogeneous with respect to race/ethnicity with 47 (46.1%) Hispanic, 28 (27.5%) White non-Hispanic, 6 (5.9%) Black non-Hispanic, 2 (2.0%) Asian/Pacific Islander, 1 (1.0%) American Indian or Alaskan Native, 14 (13.7%) other, and 4 (3.9%) missing. Mean socioeconomic status (SES) was 36.46 (SD = 16.05), based on the Hollingshead index, indicating on average a lower middle-class family SES [47].

### Measures

#### The PedsQL™ 4.0 (Pediatric Quality of Life Inventory™ Version 4.0)

The 23-item PedsQL™ 4.0 Generic Core Scales encompass: 1) Physical Functioning (8 items), 2) Emotional Functioning (5 items), 3) Social Functioning (5 items), and 4) School Functioning (5 items), and were developed through focus groups, cognitive interviews, pre-testing, and field testing measurement development protocols [28,51]. The instrument takes approximately 5 minutes to complete [51].

The PedsQL™ 4.0 Generic Core Scales are comprised of parallel child self-report and parent proxy-report formats. Child self-report includes ages 5–7, 8–12, and 13–18 years. Parent proxy-report includes ages 2–4 (toddler), 5–7 (young child), 8–12 (child), and 13–18 (adolescent), and assesses parent's perceptions of their child's HRQOL. The items for each of the forms are essentially identical, differing in developmentally appropriate language, or first or third person tense. The instructions ask how much of a problem each item has been during the past one month. A 5-point response scale is utilized across child self-report for ages 8–18 and parent proxy-report (0 = never a problem; 1 = almost never a problem; 2 = sometimes a problem; 3 = often a problem; 4 = almost always a problem).

Items are reverse-scored and linearly transformed to a 0–100 scale (0 = 100, 1 = 75, 2 = 50, 3 = 25, 4 = 0), so that higher scores indicate better HRQOL. Scale Scores are computed as the sum of the items divided by the number of items answered (this accounts for missing data). If more than 50% of the items in the scale are missing, the Scale Score is not computed. This accounts for the differences in sample sizes for scales reported in the Tables. Although there are other strategies for imputing missing values, this computation is consistent with the previous PedsQL™ peer-reviewed publications, as well as other well-established HRQOL measures [51-53]. The Physical Health Summary Score (8 items) is the same as the Physical Functioning Scale. To create the Psychosocial Health Summary Score (15 items), the mean is computed as the sum of the items divided by the number of items answered in the Emotional, Social, and School Functioning Scales.

#### PedsQL™ Multidimensional Fatigue Scale

The 18-item PedsQL™ Multidimensional Fatigue Scale encompasses: 1) General Fatigue (6 items, e.g., "I feel tired."; "I feel too tired to do things that I like to do."), 2) Sleep/Rest Fatigue (6 items, e.g., "I feel tired when I wake up in the morning."; "I rest a lot."), and 3) Cognitive Fatigue (6 items, e.g., "It is hard for me to keep my attention on things."; "It is hard for me to remember what people tell me."), with demonstrated reliability and validity in pediatric rheumatology [46]. The format, instructions, Likert scale, and scoring method are identical to the PedsQL™ 4.0 Generic Core Scales, with higher scores indicating better HRQOL (fewer problems or symptoms).

#### PedsQL™ Rheumatology Module Pain and Hurt Scale

The 22-item multidimensional PedsQL™ 3.0 Rheumatology Module includes a 4-item Pain and Hurt Scale (e.g., "I ache or hurt in my joints and/or muscles," "I feel stiff in the morning or when I sit too long"), with demonstrated reliability and validity in pediatric rheumatology [45]. The format, instructions, Likert scale, and scoring method are identical to the PedsQL™ 4.0 Generic Core Scales, with higher scores indicating better HRQOL (fewer problems or symptoms).

#### PedsQL™ Family Information Form

The PedsQL™ Family Information Form [51] was completed by parents. The PedsQL™ Family Information Form contains demographic information including the child's date of birth, gender, race/ethnicity, and parental education and occupation information required to calculate the Hollingshead four factor socioeconomic status (SES) index [47].

### Statistical analyses

The feasibility of the PedsQL™ Scales as outcome measures for pediatric patients with FM was determined from the percentage of missing values for each item [52]. Scale internal consistency reliability was determined by calculating Cronbach's coefficient alpha [54]. Scales with reliabilities of 0.70 or greater are recommended for comparing patient groups, while a reliability criterion of 0.90 is recommended for analyzing individual patient scale scores [55,56].

Construct validity was determined utilizing the known-groups method. The known-groups method compares scale scores across groups known to differ in the health construct being investigated [52,57]. In this study, analysis of variance with Tukey post-hoc tests was used to compare groups differing in known health status (pediatric patients with FM and healthy children) on the PedsQL™ 4.0 Generic Core Scales and the PedsQL™ Multidimensional Fatigue Scale. We also explored comparisons between the Generic Core Scales and Multidimensional Fatigue Scale for pediatric patients with FM with pediatric patients with cancer on-treatment and pediatric patients with other rheumatologic conditions. We further compared pain reports between pediatric patients with FM with pediatric patients with other rheumatologic conditions using independent sample *t*-tests. In order to determine the magnitude of the differences between pediatric patients with FM and healthy children, effect sizes were calculated [58]. Effect size as utilized in these analyses was calculated by taking the difference between the healthy sample mean and the FM sample mean, divided by the healthy sample standard deviation. Effect sizes for differences in means are designated as small (.20), medium (.50), and large (.80) in magnitude [58].

Construct validity for the PedsQL™ Scales in FM was further examined through an analysis of Pearson's Product Moment Correlations among the Generic Core Scales and summary scores with the pain and fatigue scales. Based on the conceptualization of disease-specific symptoms as causal indicators of generic HRQOL, and the extant literature on adult patients with FM, it was anticipated that more severe pain and fatigue would be associated with more impaired generic HRQOL. Computing the intercorrelations among scales provides additional information on the construct validity of an instrument [56]. Pearson's Product Moment Correlation coefficient effect sizes are designated as small (.10–.29), medium (.30–.49), and large (≥ .50) [58]. Intercorrelations were expected to demonstrate medium to large effect sizes.

Agreement between child self-report and parent proxy-report was determined through two-way mixed effect model (absolute agreement, single measure) Intraclass Correlations (ICC) [59]. The ICC offers an index of absolute agreement given that it takes into account the ratio between subject variability and total variability [59,60]. Intraclass Correlations are designated as ≤ 0.40 poor to fair agreement, 0.41–0.60 moderate agreement, 0.61–0.80 good agreement, and 0.81–1.00 excellent agreement [61,62]. Statistical analyses were conducted using SPSS Version 14.0 for Windows [48].

## Results

### Feasibility

To assess instrument feasibility, the percentage of missing values was calculated. The overall percentage of missing item responses across the PedsQL™ scales for patient self-report and parent proxy-report for the FM sample was 0.53% and 0.70%, respectively. For patient self-report and parent proxy-report, the percentage of missing item responses for the FM sample on the PedsQL™ 4.0 Generic Core Scales was 0.40% and 0.96%, respectively. For patient self-report and parent proxy-report, the percentage of missing item responses for the FM sample on the PedsQL™ Multidimensional Fatigue Scale was 0.98% and 0.27%, respectively. For patient self-report and parent proxy-report, the percentage of missing item responses for the FM sample on the PedsQL™ Rheumatology Module Pain and Hurt scale was 0.44% and 0.00%, respectively.

### Internal consistency reliability

Internal consistency reliability alpha coefficients for the PedsQL™ 4.0 Generic Scales and summary scores are presented in Table 1. Internal consistency reliability alpha coefficients for the PedsQL™ Multidimensional Fatigue scales and the PedsQL™ Rheumatology Module Pain and Hurt scale are presented in Table 2. On the PedsQL™ 4.0 Generic Core Scales and Multidimensional Fatigue Scale, all the patient self-report scales and parent proxy-report scales met or exceeded the minimum reliability standard of 0.70 required for group comparisons, while the Generic Core Total Scale Score and Total Fatigue Scale Score for both child self-report and parent proxy-report approached or exceeded the reliability criterion of 0.90 recommended for analyzing individual patient scale scores. On the PedsQL™ Rheumatology Module Pain and Hurt Scale, the child self-report and parent proxy-report alpha coefficients approached or exceeded the minimum reliability standard of 0.70 required for group comparisons.

**Table 1:** PedsQL™ 4.0 Generic Core Scales Scores for Child Self-Report and Parent Proxy-Report across Samples and Reliability for Fibromyalgia Sample

	Fibromyalgia^a^			Cancer^b^		Rheumatology^c^		Healthy^d^		Differences	Effect Size
Scale	α	Mean	SD	Mean	SD	Mean	SD	Mean	SD		

**Self-Report**	**(N = 56)**	**(N = 57)**		**(N = 74)**		**(N = 154)**		**(N = 2873)**			
Total Score	0.88	55.85	15.16	68.48	15.25	76.01	15.88	82.70	13.20	a<b,c,d***	2.03
Physical Functioning	0.85	45.40	19.86	63.03	23.90	71.72	22.24	86.29	13.69	a<b,c,d***	2.99
Psychosocial Health	0.84	61.45	16.25	71.78	13.79	78.33	14.57	80.80	14.55	a<b,c,d***	1.33
Emotional Functioning	0.78	53.77	22.41	67.91	18.60	75.75	19.21	76.60	18.72	a<b,c,d***	1.22
Social Functioning	0.70	74.47	18.29	79.70	17.73	84.67	16.27	85.27	17.15	a<c**; a<d***	0.63
School Functioning	0.74	55.74	20.46	67.61	19.37	75.07	17.44	80.51	16.13	a<b,c,d***	1.54
**Proxy-Report**	**(N = 56)**	**(N = 57)**		**(N = 78)**		**(N = 131)**		**(N = 3371)**			
Total Score	0.87	52.04	15.17	58.23	19.24	73.41	17.47	78.78	16.58	a<c,d***	1.61
Physical Functioning	0.76	43.37	19.01	53.22	27.11	69.07	23.66	80.53	21.11	a<b*; a<c,d***	1.76
Psychosocial Health	0.85	56.70	16.41	61.25	17.84	75.73	16.73	77.82	16.39	a<c,d***	1.29
Emotional Functioning	0.76	48.95	19.47	57.12	22.34	72.56	20.38	77.71	17.76	a<b*; a<c,d***	1.62
Social Functioning	0.71	67.02	19.70	70.24	19.42	79.84	19.72	80.42	20.88	a<c**; a<d***	0.64
School Functioning	0.75	53.77	20.55	57.35	22.20	75.15	19.90	75.32	20.14	a<c,d***	1.07

*p < .05, **p < .01, ***p < .001 based on Tukey post-hoc analysis. Effect sizes are designated as small (.20), medium (.50), and large (.80). Effect sizes represent the magnitude in the differences between the Fibromyalgia and Healthy Children samples only. α = Cronbach internal consistency reliability coefficient alpha.

**Table 2:** PedsQL™ Multidimensional Fatigue Scale and Rheumatology Module Pain and Hurt Scale for Child Self-Report and Parent Proxy-Report across Samples and Reliability for Fibromyalgia Sample

	Fibromyalgia^a^			Cancer^b^		Rheumatology^c^		Healthy^d^		Differences	Effect Size
Scale	α	Mean	SD	Mean	SD	Mean	SD	Mean	SD		

**Self-Report**	**(N = 28)**	**(N = 29)**		**(N = 75)**		**(N = 152)**		**(N = 52)**			
Total Fatigue	0.94	55.48	21.19	70.23	15.68	76.68	20.52	80.49	13.33	a<b**; a<c,d***	1.88
General Fatigue	0.91	48.97	25.14	71.69	19.42	76.82	23.19	85.34	14.95	a<b,c,d***	2.43
Sleep/Rest Fatigue	0.76	52.36	21.08	64.50	21.73	71.77	24.27	75.00	18.76	a<c,d***	1.21
Cognitive Fatigue	0.91	65.17	24.30	74.49	19.10	81.30	20.83	81.14	17.43	a<c,d**	0.92
Pain and Hurt Scale	0.68	32.93	26.88	NA		67.11	27.54	NA		a<c***	NA
**Proxy-Report**	**(N = 26)**	**(N = 26)**		**(N = 79)**		**(N = 125)**		**(N = 102)**			
Total Fatigue	0.94	54.19	18.99	64.05	19.08	75.78	20.81	89.63	11.38	a<c,d***	3.11
General Fatigue	0.89	49.68	21.92	60.04	23.39	73.11	24.65	89.30	13.33	a<c,d***	2.97
Sleep/Rest Fatigue	0.84	48.85	21.30	56.97	24.37	72.86	23.85	88.86	14.72	a<c,d***	2.72
Cognitive Fatigue	0.96	64.10	22.70	75.14	21.37	81.83	19.39	90.72	15.15	a<c,d***	1.76
Pain and Hurt Scale	0.75	32.35	25.93	NA		65.33	28.96	NA		a<c***	NA

*p < .05, **p < .01, ***p < .001 based on Tukey post-hoc analysis. Effect sizes are designated as small (.20), medium (.50), and large (.80). Effect sizes represent the magnitude in the differences between the Fibromyalgia and Healthy Children samples only. α = Cronbach internal consistency reliability coefficient alpha. Healthy sample completed the Acute Version (7-day recall) of the PedsQL™ Multidimensional Fatigue Scale. Sample size for the Rheumatology Module Pain and Hurt Scale for Child Self-Report for Fibromyalgia sample equals 57 and for Rheumatology sample equals 150. Sample size for the Rheumatology Module Pain and Hurt Scale for Parent Proxy-Report for Fibromyalgia sample equals 57 and for Rheumatology sample equals 126. NA = not applicable.

### Construct validity

Tables 1 and 2 present the means and standard deviations of the PedsQL™ 4.0 Generic Core Scales and Multidimensional Fatigue Scale scores for patient self-report and parent proxy-report for pediatric patients with FM and healthy children. For all PedsQL™ 4.0 Generic Core Scales and summary scores, pediatric patients with FM and their parents reported statistically significant lower HRQOL than healthy children. Pediatric patients with FM and their parents also reported significantly worse fatigue than healthy children. Most effect sizes were in the large range.

### Comparison to pediatric cancer and pediatric rheumatology

Tables 1 and 2 also present the means and standard deviations of the PedsQL™ 4.0 Generic Core Scales and Multidimensional Fatigue Scale scores for patient self-report and parent proxy-report for pediatric patients with cancer on-treatment and pediatric patients with rheumatologic conditions other than FM. Table 1 demonstrates that across the PedsQL™ 4.0 Generic Core Scales and summary scores, with the exception of the Social Functioning Scale, children with FM in this sample reported statistically significant lower HRQOL than children with cancer on-treatment and children with a variety of rheumatologic conditions. In addition, pediatric patients with FM reported significantly worse total fatigue and general fatigue than children with cancer on-treatment and significantly worse fatigue across all the fatigue dimensions than pediatric patients with other rheumatologic conditions. Table 2 also presents the means and standard deviations of the PedsQL™ 3.0 Rheumatology Module Pain and Hurt Scale between pediatric patients with FM and pediatric patients with other rheumatologic conditions. Pediatric patients with FM and their parents reported significantly worse pain than pediatric patients with other rheumatologic conditions and their parents.

### Intercorrelations among PedsQL™ Scales

Table 3 shows the intercorrelations among the Generic Core Scales and summary scores with the pain and fatigue scales. More severe pain and fatigue was significantly correlated with more impaired generic HRQOL. These intercorrelations are in the large effect size range.

**Table 3:** Intercorrelations among PedsQL™ Scales for Child Self-Report and Parent Proxy-Report for Fibromyalgia Sample

Scale	TG	PF	PH	EF	SF	SchF	TF	GF	S/RF	CF	P/HS
Total Generic Core (TG)	0.63***	0.79***	0.92***	0.71***	0.68***	0.81***	0.81***	0.81***	0.67**	0.69***	0.72***
Physical Functioning (PF)	0.80***	0.47***	0.48***	0.27*	0.40**	0.49***	0.75***	0.74***	0.61**	0.64**	0.63***
Psychosocial Health (PH)	0.93***	0.51***	0.66***	0.83***	0.71***	0.84***	0.69***	0.69***	0.57**	0.58**	0.62***
Emotional Functioning (EF)	0.73***	0.44**	0.76***	0.66***	0.35**	0.58***	0.50*	0.49*	0.31	0.49*	0.51***
Social Functioning (SF)	0.80***	0.45***	0.85***	0.45**	0.49***	0.40**	0.45*	0.47*	0.58**	0.21	0.43**
School Functioning (SchF)	0.77***	0.38**	0.85***	0.44**	0.65***	0.59***	0.71***	0.68***	0.47*	0.73***	0.54***
Total Fatigue (TF)	0.75***	0.61**	0.62**	0.53*	0.40	0.63**	0.81***	0.93***	0.84***	0.90***	0.64**
General Fatigue (GF)	0.77***	0.70***	0.60**	0.55**	0.38	0.52*	0.89***	0.78***	0.72***	0.76***	0.67***
Sleep/Rest Fatigue (S/RF)	0.53*	0.39	0.46*	0.41	0.33	0.38	0.84***	0.74***	0.77***	0.62**	0.48*
Cognitive Fatigue (CF)	0.59**	0.44*	0.50*	0.38	0.30	0.71***	0.80***	0.54*	0.45*	0.75***	0.53**
Pain and Hurt Scale (P/HS)	0.64***	0.53***	0.58***	0.56***	0.42**	0.44**	0.63**	0.66**	0.63**	0.34	0.73***

Pearson's product moment correlations for patient self-report above diagonal, Pearson's product moment correlations for parent proxy-report below diagonal, and two-way mixed effect model (absolute agreement, single measure) Intraclass Correlations (ICCs) for patient/parent agreement on diagonal. Effect sizes are designated as small (0.10), medium (0.30), and large (0.50) for Pearson's product moment correlations. ICCs are designated as ≤ 0.40 poor to fair agreement, 0.41–0.60 moderate agreement, 0.61–0.80 good agreement, and 0.81–1.00 excellent agreement. *p < .05, **p <. 01, ***p < .001.

### Parent/child agreement

Table 3 presents two-way mixed effect model (absolute agreement, single measure) Intraclass Correlations (ICC) between pediatric patients with FM self-report and parent proxy-report across the PedsQL™ scales. Most ICCs are in the range of moderate to good agreement, with the greatest overall agreement on the fatigue and pain scales. On the PedsQL™ 4.0 Generic Core Scales, the greatest agreement is on the Psychosocial Health Summary Score and Emotional Functioning Scale.

## Discussion

These analyses demonstrate the feasibility, reliability and validity of the PedsQL™ as a multidimensional pediatric patient-reported outcome instrument for fibromyalgia consistent with the OMERACT Fibromyalgia Syndrome Workshop recommendations [4]. These PedsQL™ Scales measure the OMERACT prioritized domains that should be consistently measured in FM clinical trials, specifically, pain, generic HRQOL, fatigue, sleep quality, and physical function [4]. In addition, in adult patients with FM, recent evidence suggests the importance of measuring cognitive difficulties ("fibrofog"), frequently reported by adult patients with FM as memory and attention problems [3]. The PedsQL™ Cognitive Fatigue Scale measures the construct of cognitive problems as delineated in the adult FM literature, and to our knowledge, represents the first measurement presentation of this construct in pediatric patients with FM.

Items on the PedsQL™ Scales had minimal missing responses, suggesting that pediatric patients with FM and their parents are willing and able to provide good quality data regarding the patient's HRQOL. The PedsQL™ self-report and proxy-report internal consistency reliabilities generally exceeded the recommended minimum alpha coefficient standard of 0.70 for group comparisons. The PedsQL™ Generic Core Scales Total Score and Multidimensional Fatigue Scale Total Score for pediatric patient self-report and parent proxy-report approached or exceeded an alpha of 0.90, recommended for individual patient analysis [55], making the Generic Core Scales Total Scale Score suitable as a summary score for the primary analysis of HRQOL outcome in clinical trial analyses for pediatric patients with FM, with the PedsQL™ Psychosocial Health Summary Score, the Multidimensional Fatigue Scale Total Score, and individual scales suitable alternatively as either the primary or secondary outcome score depending on the intent of a particular clinical trial.

As hypothesized, pediatric patients with FM self-reported significantly lower PedsQL™ scores on dimensions of physical and psychosocial health and fatigue in comparison to healthy children. These findings are consistent with findings in adult patients with FM using the SF-36 [13-15]. We believe the consistency of the present findings in which differences between healthy children and pediatric patients with FM for *both *child self-report and parent proxy-report support the magnitude of the reported impairment in these pediatric patients with FM. This illustrates the benefits of the PedsQL™ Measurement Model in which both child self-report and parent proxy-report are measured [28].

The comparisons between pediatric patients with FM with pediatric cancer patients receiving cancer treatment and those pediatric patients with other rheumatologic diseases are useful in understanding the relative clinical impact of fibromyalgia on HRQOL. The extant literature on the adaptation of children with chronic physical health conditions demonstrates that children with chronic physical health conditions are reported to not only experience lower physical functioning, but also manifest lower emotional, social, and school functioning in comparison to healthy children [63]. The findings that pediatric patients with FM report physical and psychosocial health generally lower than children receiving chemotherapy and radiation for the treatment of pediatric cancer provide further insight into the comparative negative impact of this chronic health condition on HRQOL in comparison to other serious pediatric chronic conditions. The findings that pediatric patients with FM, whose examinations and laboratory tests are generally normal, demonstrated significantly lower overall HRQOL and more severe pain and fatigue than pediatric patients with other rheumatologic conditions, whose examinations and tests are generally abnormal, further emphasizes the importance of measuring HRQOL outcomes in these children and adolescents.

These findings with the PedsQL™ Scales have potential clinical implications for the healthcare needs of children with FM. Given the degree of reported impairment in their HRQOL, the urgent need for efficacious treatments is quite evident. The immediate and long-term consequences of untreated or under-treated FM appears quite severe for these children, their families, and society as a whole [7,64]. The challenge for health care is to identify and enroll pediatric patients with FM in high quality evidence-based comprehensive healthcare services in order to mitigate the potential long-term negative consequences on patient HRQOL. For chronic health conditions such as fibromyalgia, HRQOL and symptom scales completed by patients may be the only indicators of disease activity and treatment effect [21-23]. In such patient populations, PROs are often indicated as the primary end-points for drug approval [21-23]. Given that pharmaceutical and nonpharmaceutical treatment regimens for adults with FM are emerging [65-68], trials which evaluate the impact of similar regimens on the patient-reported health outcomes of pediatric patients with FM are urgently needed [7,64,69].

Finally, while self-report is considered the standard for measuring perceived HRQOL, it is typically parents' perceptions of their children's HRQOL that influences healthcare utilization [37-39]. Thus, the imperfect agreement observed between child self-report and parent proxy-report supports the need to measure the perspectives of both the child and parent in evaluating pediatric HRQOL since these perspectives may be independently related to healthcare utilization and risk factors. The availability of a validated parent proxy-report measure in FM provides the opportunity to estimate child HRQOL when the child is either unable or unwilling to complete the HRQOL measure. Although the intercorrelations between child and parent report across the physical, psychosocial, fatigue and pain domains might be expected to follow the conceptualization that more observable domains (i.e., physical functioning) would yield higher intercorrelations, this has not necessarily been the case in either PedsQL™ publications across various pediatric chronic health conditions, or in the published literature with other HRQOL instruments. In a comprehensive review, Eiser [70] found mixed results in terms of higher intercorrelations between self and proxy report of physical functioning across pediatric HRQOL instruments, with most studies demonstrating this effect, while some others did not. In a condition such as FM that is highly associated with significant pain, fatigue, and emotional distress, parents may be more acutely attuned to these symptoms, and consequently their perception of their child's HRQOL on these domains may more closely align with their child's perceptions.

The present findings have several potential limitations. Given that the comparative analyses were with an exiting database, we were only able to match the FM sample to the pediatric cancer and rheumatology samples by age, not gender, given the small sample sizes for these chronic health conditions. Other sociodemographic differences of these existing databases may have further influenced the comparative findings with healthy children, and pediatric patients with cancer and other rheumatologic conditions; however, our findings are consistent with the literature on adult patients with FM. Further, the PedsQL™ Multidimensional Fatigue Scale was not administered initially in our FM recruitment efforts, and thus we report a smaller sample size for analyses here. Additionally, sensitivity and responsiveness data were not available for these analyses; however, previous PedsQL™ research with patients with rheumatologic and other chronic health conditions have demonstrated the sensitivity and responsiveness of the PedsQL™ Scales [17,20,45,71-73]. Finally, we have no information on nonparticipants given the requirements of the local IRB.

## Conclusion

These PedsQL™ findings demonstrate the feasibility and measurement properties required for pediatric clinical trials and health services research in pediatric patients with fibromyalgia. As a chronic musculoskeletal pain syndrome with no identifiable cause and no physiological markers of disease activity, FM represents a significant challenge in the clinical decision-making process. The treating physician cannot monitor the course of the illness with objective disease indicators that are available for other rheumatologic diseases, and thus is dependent on patient self-report. These PedsQL™ Scales provide a measurement approach that has great potential as outcome measures in the clinical setting in this regard. Further, pediatric patient-reported outcomes should be considered as the standard for HRQOL measurement in pediatric clinical trials in which patient health-related quality of life is investigated in FM. In this way, the voices of the children will be heard in matters pertaining to their health and well-being given the perspective that "some treatment effects are known only to the patient" [12]. Measuring perceived health from the perspective of pediatric patients with fibromyalgia provides a level of accountability consistent with the Institute of Medicine report on the quality of care [74]. As the consumers of pediatric healthcare, children and their parents are uniquely positioned to give their perspectives on healthcare quality through their perceptions of patient health-related quality of life.

## Abbreviations

HRQOL Health-Related Quality of Life

PedsQL™ Pediatric Quality of Life Inventory™

PRO Patient-Reported Outcomes

FM Fibromyalgia

OMERACT Outcome Measures in Rheumatoid Arthritis Clinical Trials

## Competing interests

Dr. Varni holds the copyright and the trademark for the PedsQL™ and receives financial compensation from the Mapi Research Trust, which is a nonprofit research institute that charges distribution fees to for-profit companies that use the Pediatric Quality of Life Inventory™. The PedsQL™ is available at the PedsQL™ Website [75].

## Authors' contributions

JWV conceptualized the rationale and design of the study. JWV and CAL drafted the manuscript. CAL performed the statistical analyses. TMB participated in the conceptualization of the study, in drafting the manuscript and the statistical analyses. ISS participated in the conceptualization of the study and patient recruitment. All authors read and approved the final manuscript.

## References

[B1] Wolfe F, Smythe HA, Yunus MB, Bennett RM, Bombardier C, Goldenberg DL, Tugwell P, Campbell SM, Abeles M (1990). The American College of Rheumatology 1990 criteria for the classification of fibromyalgia: Report of the multicenter criteria committee. Arthritis and Rheumatism.

[B2] Geenen R, Jacobs JW (2001). Fibromyalgia: Diagnosis, pathogenesis, and treatment. Current Opinion in Anaesthesiology.

[B3] Katz RS, Heard AR, Mills M, Leavitt F (2004). The prevalence and clinical impact of reported cognitive difficulties (fibrofog) in patients with rheumatic disease with and without fibromyalgia. Journal of Clinical Rheumatology.

[B4] Mease PJ, Clauw DJ, Arnold LM, Goldenberg DL, Witter J, Williams DA, Simon LS, V SC, Bramson C, Martin S, Wright TM, Littman B, Wernicke JF, Gendreau RM, Crofford LJ (2005). Fibromyalgia syndrome. Journal of Rheumatology.

[B5] Yunus MB, Masi AT (1985). Juvenile primary fibromyalgia syndrome: A clinical study of thirty-three patients and matched normal controls. Arthritis and Rheumatism.

[B6] Wolfe F, Ross K, Anderson J, Russell IJ, Hebert L (1995). The prevalence and characteristics of fibromyalgia in the general population. Arthritis and Rheumatism.

[B7] Kashikar-Zuck S, Graham TB, Huenefeld MD, Powers SW (2000). A review of biobehavioral research in juvenile primary fibromyalgia syndrome. Arthritis Care and Research.

[B8] Fayers PM, Machin D (2000). Quality of life: Assessment, analysis, and interpretation.

[B9] Spilker B (1996). Quality of life and pharmacoeconomics in clinical trials.

[B10] Varni JW, Seid M, Kurtin PS (1999). Pediatric health-related quality of life measurement technology: A guide for health care decision makers. Journal of Clinical Outcomes Management.

[B11] World Health Organization (1948). Constitution of the World Health Organization: Basic Document.

[B12] FDA (2006). Guidance for Industry: Patient-reported outcome measures: Use in medical product development to support labeling claims.

[B13] Bennett RM, Schein J, Kosinski MR, Hewitt DJ, Jordan DM, Rosenthal NR (2005). Impact of fibromyalgia pain on health-related quality of life before and after treatment with tramadol/acetaminophen. Arthritis and Rheumatism.

[B14] Birtane M, Uzunca K, Tastekin N, Tuna H (2006). The evaluation of quality of life in fibromyalgia syndrome: A comparison with rheumatoid arthritis by using SF-36 health survey. Clinical Rheumatology.

[B15] Strombeck B, Ekdahl C, Manthorpe R, Wikstrom I, Jacobsson L (2000). Health-related quality of life in primary Sjogren's syndrome, rheumatoid arthritis and fibromyalgia compared to normal population data using SF-36. Scandinavian Journal of Rheumatology.

[B16] Eiser C (2004). Use of quality of life measures in clinical trials. Ambulatory Pediatrics.

[B17] Razzouk BI, Hord JD, Hockenberry M, Hinds PS, Feusner J, Williams D, Rackoff WR (2006). Double-blind, placebo-controlled study of quality of life, hematologic end points, and safety of weekly epoetin alfa in children with cancer receiving myelosuppressive chemotherapy. Journal of Clinical Oncology.

[B18] Varni JW, Burwinkle TM, Seid M (2005). The PedsQL™ as a pediatric patient-reported outcome: Reliability and validity of the PedsQL™  Measurement Model in 25,000 children. Expert Review of Pharmacoeconomics and Outcomes Research.

[B19] Schwimmer JB, Middleton MS, Deutsch R, Lavine JE (2005). A phase 2 trial of metformin as a treatment for non-diabetic pediatric non-alcoholic steatohepatitis. Alimentary Pharmacology & Therapeutics.

[B20] Connelly M, Rapoff MA (2006). Assessing health-related quality of life in children with recurrent headache: Reliability and validity of the PedsQL™ 4.0 in a pediatric sample. Journal of Pediatric Psychology.

[B21] Acquadro C, Berzon R, Dubois D, Leidy NK, Marquis P, Revicki D, Rothman M (2003). Incorporating the patient's perspective into drug development and communication: An ad hoc task force report of the patient-reported outcomes (PRO) harmonization group meeting at the Food and Drug Administration, February 16, 2001. Value in Health.

[B22] Willke RJ, Burke LB, Erickson P (2004). Measuring treatment impact: A review of patient-reported outcomes and other efficacy endpoints in approved product labels. Controlled Clinical Trials.

[B23] Sherman SA, Eisen S, Burwinkle TM, Varni JW (2006). The PedsQL™ Present Functioning Visual Analogue Scales: Preliminary reliability and validity. Health and Quality of Life Outcomes.

[B24] Sprangers MAG, Aaronson NK (1992). The role of health care providers and significant others in evaluating the quality of life of patients with chronic disease: A review. Journal of Clinical Epidemiology.

[B25] Achenbach TM, McConaughy SH, Howell CT (1987). Child/adolescent behavioral and emotional problems: Implications of cross-informant correlations for situational specificity. Psychological Bulletin.

[B26] Varni JW, Katz ER, Colegrove R, Dolgin M (1995). Adjustment of children with newly diagnosed cancer: Cross-informant variance. Journal of Psychosocial Oncology.

[B27] Varni JW, Katz ER, Seid M, Quiggins DJL, Friedman-Bender A, Castro CM (1998). The Pediatric Cancer Quality of Life Inventory (PCQL): I.  Instrument development, descriptive statistics, and cross-informant variance. Journal of Behavioral Medicine.

[B28] Varni JW, Seid M, Rode CA (1999). The PedsQL™: Measurement model for the Pediatric Quality of Life Inventory. Medical Care.

[B29] Levi RB, Drotar D (1999). Health-related quality of life in childhood cancer: Discrepancy in parent–child reports. International Journal of Cancer.

[B30] Clancy C, McGrath P, Oddson B (2005). Pain in children and adolescents with spina bifida. Developmental Medicine and Child Neurology.

[B31] Felder-Puig R, diGallo A, Waldenmair M, Norden P, Winter A, Gadner H, Topf R (2006). Health-related quality of life of pediatric patients receiving allogeneic stem cell or bone marrow transplantation: Results of a longitudinal, multi-center study. Bone Marrow Transplantation.

[B32] Vance YH, Morse RC, Jenney ME, Eiser C (2001). Issues in measuring quality of life in childhood cancer: Measures, proxies, and parental mental health. Journal of Child Psychology and Psychiatry.

[B33] Chang P, Yeh C (2005). Agreement between child self-report and parent proxy-report to evaluate quality of life in children with cancer. Psycho-Oncology.

[B34] Yeh CH, Chang CW, Chang PC (2005). Evaluating quality of life in children with cancer using children's self-reports and parent-proxy reports. Nursing Research.

[B35] Varni JW, Limbers CA, Burwinkle TM (2007). How young can children reliably and validly self-report their health-related quality of life?: An analysis of 8,591 children across age subgroups with the PedsQL™ 4.0 Generic Core Scales. Health and Quality of Life Outcomes.

[B36] Varni JW, Limbers CA, Burwinkle TM (2007). Parent proxy-report of their children’s health-related quality of life: An analysis of 13,878 parents’ reliability and validity across age subgroups using the PedsQL™ 4.0 Generic Core Scales. Health and Quality of Life Outcomes.

[B37] Campo JV, Comer DM, Jansen-McWilliams L, Gardner W, Kelleher KJ (2002). Recurrent pain, emotional distress, and health service use in childhood. Journal of Pediatrics.

[B38] Janicke DM, Finney JW, Riley AW (2001). Children's health care use: A prospective investigation of factors related to care-seeking. Medical Care.

[B39] Varni JW, Setoguchi Y (1992). Screening for behavioral and emotional problems in children and adolescents with congenital or acquired limb deficiencies. American Journal of Diseases of Children.

[B40] Varni JW, Burwinkle TM, Lane MM (2005). Health-related quality of life measurement in pediatric clinical practice: An appraisal and precept for future research and application. Health and Quality of Life Outcomes.

[B41] Cremeens J, Eiser C, Blades M (2006). Characteristics of health-related self-report measures for children aged three to eight years: A review of the literature. Quality of Life Research.

[B42] Russell KMW, Hudson M, Long A, Phipps S (2006). Assessment of health-related quality of life in children with cancer: Consistency and agreement between parent and child reports. Cancer.

[B43] Eiser C, Morse R (2001). Can parents rate their child's health-related quality of life?: Results from a systematic review. Quality of Life Research.

[B44] Varni JW, Burwinkle TM, Katz ER, Meeske K, Dickinson P (2002). The PedsQL™ in pediatric cancer: Reliability and validity of the Pediatric Quality of Life Inventory™  Generic Core Scales, Multidimensional Fatigue Scale, and Cancer Module. Cancer.

[B45] Varni JW, Seid M, Knight TS, Burwinkle TM, Brown J, Szer IS (2002). The PedsQL™ in pediatric rheumatology: Reliability, validity, and responsiveness of the Pediatric Quality of Life Inventory™ Generic Core Scales and Rheumatology Module. Arthritis and Rheumatism.

[B46] Varni JW, Burwinkle TM, Szer IS (2004). The PedsQL™ Multidimensional Fatigue Scale in pediatric rheumatology: Reliability and validity. Journal of Rheumatology.

[B47] Hollingshead AB (1975). Four factor index of social status.

[B48] SPSS (2005). SPSS 14.0 for Windows.

[B49] Varni JW, Burwinkle TM, Seid M, Skarr D (2003). The PedsQL™ 4.0 as a pediatric population health measure: Feasibility, reliability, and validity. Ambulatory Pediatrics.

[B50] Varni JW, Burwinkle TM, Seid M (2006). The PedsQL™ 4.0 as a school population health measure: Feasibility, reliability, and validity. Quality of Life Research.

[B51] Varni JW, Seid M, Kurtin PS (2001). PedsQL™ 4.0:  Reliability and validity of the Pediatric Quality of Life Inventory™ Version 4.0 Generic Core Scales in healthy and patient populations. Medical Care.

[B52] McHorney CA, Ware JE, Lu JFR, Sherbourne CD (1994). The MOS 36-item short-form health survey (SF-36): III. Tests of data quality, scaling assumptions, and reliability across diverse patient groups. Medical Care.

[B53] Fairclough DL, Cella DF (1996). Functional Assessment of Cancer Therapy (FACT-G): Non-response to individual questions. Quality of Life Research.

[B54] Cronbach LJ (1951). Coefficient alpha and the internal structure of tests. Psychometrika.

[B55] Nunnally JC, Bernstein IR (1994). Psychometric theory.

[B56] Pedhazur EJ, Schmelkin LP (1991). Measurement, design, and analysis: An integrated approach.

[B57] McHorney CA, Ware JE, Raczek AE (1993). The MOS 36-item short-form health survey (SF-36): II. Psychometric and clinical tests of validity in measuring physical and mental health constructs. Medical Care.

[B58] Cohen J (1988). Statistical power analysis for the behavioral sciences.

[B59] McGraw KO, Wong SP (1996). Forming inferences about some Intraclass Correlation Coefficients. Psychological Methods.

[B60] Cremeens J, Eiser C, Blades M (2006). Factors influencing agreement between child self-report and parent proxy-reports on the Pediatric Quality of Life Inventory™ 4.0 (PedsQL™) Generic Core Scales. Health and Quality of Life Outcomes.

[B61] Bartko JJ (1966). The intraclass correlation coefficient as a measure of reliability. Psychological Reports.

[B62] Wilson KA, Dowling AJ, Abdolell M, Tannock IF (2001). Perception of quality of life by patients, partners and treating physicians. Quality of Life Research.

[B63] Wallander JL, Varni JW (1998). Effects of pediatric chronic physical disorders on child and family adjustment. Journal of Child Psychology and Psychiatry.

[B64] Breau BA, McGrath PJ, Ju LH (1999). Review of juvenile primary fibromyalgia and chronic fatigue syndrome. Journal of Developmental and Behavioral Pediatrics.

[B65] Arnold LM (2006). Biology and therapy of fibromyalgia: New therapies in fibromyalgia. Arthritis Research and Therapy.

[B66] Lawson K (2006). Emerging pharmacological therapies for fibromyalgia. Current Opinion in Investigative Drugs.

[B67] Thieme K, Flor H, Turk DC (2006). Psychological pain treatment in fibromyalgia syndrome: Efficacy of operant behavioural and cognitive behavioural treatments. Arthritis Research and Therapy.

[B68] Garcia J, Simon MA, Duran M, Canceller J, Aneiros FJ (2006). Differential efficacy of a cognitive-behavioral intervention versus pharmacological treatment in the management of fibromyalgia syndrome. Psychological Health and Medicine.

[B69] Degotardi PJ, Klass ES, Rosenberg BS, Fox DG, Gallelli KA, Gottlieb BS (2006). Development and evaluation of a cognitive-behavioral intervention for juvenile fibromyalgia. Journal of Pediatric Psychology.

[B70] Eiser C, Morse R (2001). Quality of life measures in chronic diseases of childhood. Health Technology Assessment.

[B71] Varni JW, Seid M, Knight TS, Uzark K, Szer IS (2002). The PedsQL™ 4.0 Generic Core Scales: Sensitivity, responsiveness, and impact on clinical decision-making. Journal of Behavioral Medicine.

[B72] Varni JW, Burwinkle TM, Berrin SJ, Sherman SA, Artavia K, Malcarne VL, Chambers HG (2006). The PedsQL™ in pediatric cerebral palsy: Reliability, validity, and sensitivity of the Generic Core Scales and Cerebral Palsy Module. Developmental Medicine and Child Neurology.

[B73] Seid M, Varni JW, Cummings L, Schonlau M (2006). The impact of realized access to care on health-related quality of life: A two-year prospective cohort study of children in the California State Children’s Health Insurance Program. Journal of Pediatrics.

[B74] IOM (2001). Crossing the quality chasm: A new health system for the 21st century.

[B75] Varni JW PedsQL™ Website. http://www.pedsql.org.

